# Ant community composition and functional traits in new grassland strips within agricultural landscapes

**DOI:** 10.1002/ece3.7662

**Published:** 2021-05-25

**Authors:** Victor Sebastian Scharnhorst, Konrad Fiedler, Thomas Frank, Dietmar Moser, Dominik Rabl, Manuela Brandl, Raja Imran Hussain, Ronnie Walcher, Bea Maas

**Affiliations:** ^1^ Department of Botany and Biodiversity Research University of Vienna Vienna Austria; ^2^ Institute of Zoology University of Natural Resources and Life Sciences (BOKU) Vienna Austria

**Keywords:** agricultural biodiversity, ant community composition, Austria, biocontrol, ecosystem services, functional traits

## Abstract

1. Ongoing intensification and fragmentation of European agricultural landscapes dramatically reduce biodiversity and associated functions. Enhancing perennial noncrop areas holds great potential to support ecosystem services such as ant‐mediated pest control.
2. To study the potential of newly established grassland strips to enhance ant diversity and associated functions, we used hand collection data and predation experiments to investigate differences in (a) ant community composition and (b) biocontrol‐related functional traits, and (c) natural pest control across habitats in cereal fields, old grasslands, and new grassland transects of three years of age.
3. Ant species diversity was similar between new and old grasslands, but significantly higher in new grasslands than in surrounding cereal fields. Contrary, ant community composition of new grasslands was more similar to cereal fields and distinct from the species pool of old grasslands. The functional trait space covered by the ant communities showed the same distribution between old and new grasslands. Pest control did not differ significantly between habitat types and therefore could not be linked to the prevalence of functional ant traits related to biocontrol services in new grasslands.
4. Our findings not only show trends of convergence between old and new grasslands, but also indicate that enhancing ant diversity through new grasslands takes longer than three years to provide comparable biodiversity and functionality.
5. *Synthesis and applications*: Newly established grasslands can increase ant species richness and abundance and provide a consistent amount of biocontrol services in agroecosystems. However, three years after their establishment, new grasslands were still dominated by common agrobiont ant species and lacked habitat specialists present in old grasslands, which require a constant supply of food resources and long colony establishment times. New grasslands represent a promising measure for enhancing agricultural landscapes but must be preserved in the longer term to promote biodiversity and resilience of associated ecosystem services.

## Graphical abstract

Newly established grasslands can increase ant species richness, abundance and provide a consistent amount of biocontrol services in agroecosystems. However, three years after their establishment, new grasslands were still dominated by common agrobiont ant species and lacked habitat specialists present in old grasslands, which require a constant supply of food resources and long colony establishment times. New grasslands represent a promising measure for enhancing agricultural landscapes but must be preserved in the longer term to promote biodiversity and resilience of associated ecosystem services.

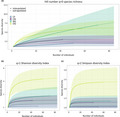

## 1 INTRODUCTION

European countries are spatially dominated by agricultural landscapes (Kleijn et al., 2012), yet the ongoing intensification of their management dramatically reduces biodiversity (Cardoso et al., 2020). Species diversity and specific ecological traits are well known as key promoters of ecosystem functioning (Borer et al., 2017). However, ubiquitous ecosystem engineers, such as ants (Sanders & van Veen, 2011), are threatened by destruction and fragmentation of remaining semi‐natural habitats interspersed between arable lands (Ewers & Didham, 2006; Hendrickx et al., 2007). To mitigate severe effects on the maintenance of ecosystem services provided by ants, such as biological pest control (Tscharntke et al., 2012), biodiversity restoration in modern cultivated landscapes holds great potential (Ekroos et al., 2014; Tscharntke et al., 2005). Extensively managed grassland ecosystems are among the most species‐rich habitats in northern and central Europe and paramount for the diversity of ants in temperate regions (Seifert, 2018). These important semi‐natural environments disappear rapidly from European agricultural landscapes due to abandonment, afforestation, and conversion to residential areas (Valkó et al., 2018). Set‐aside land and other remnants of semi‐natural habitats enhance the edge density in agricultural landscapes and foster the diversity, abundance, and functionality of ground‐dwelling predators such as ants (Martin et al., 2019). However, yield‐enhancing ecosystem services that are provided in farmland areas rely heavily on the ability of predator species to disperse into the agricultural matrix (Kohler et al., 2008). Enhancing perennial noncrop areas through newly established grassland strips (hereafter new grasslands) likely provides refuge habitat for both common and more specialized agrobiont species, if they persist in the long term (Dauber & Wolters, 2005).

Ants are eusocial insects and important consumers of herbivorous insects, making them a key taxon for ecosystem functioning of temperate grasslands (Wills & Landis, 2018). Many ant species can organize mass recruitment of nest mates if sufficient food sources are available (Seifert, 2018), and this spatial allocation of predatory workers enables ant colonies to respond effectively to a dynamic and heterogeneous density of prey in their environment (Way & Khoo, 1992). Yet, the importance of ants as consumers and ecosystem engineers is often underappreciated (Wills & Landis, 2018), even though they are the numerically dominant invertebrates in certain agricultural landscapes. Despite other important ecological functions such as litter recycling and seed dispersal (Seifert, 2018), ants are important predators of pest lepidopteran and coleopteran larvae in agricultural landscapes (Wills & Landis, 2018). Furthermore, Offenberg (2015) showed that the efficiency of ant‐mediated biocontrol is comparable to chemical pesticides, which persistently reduce the opportunity for biological pest control in farmlands (Geiger et al., 2010). Therefore, pest control provided by ants constitutes an alternative to pesticide application and designates ants as a relevant target group toward the development of sustainable management practices of agroecosystems.

Similar to most other grassland taxa, ants are highly responsive to human impact, such as land‐use change (Dauber & Wolters, 2005). A recent study highlights that ant species richness, as well as functional diversity of ant communities, decreases with increasing land‐use intensity in terms of mowing and grazing of grasslands (Heuss et al., 2019). However, to maintain ant biodiversity and their role as biocontrol agents, not only the underlying mechanisms leading to the aforementioned decreases have to be elucidated, but also how habitat restoration, in terms of new grasslands, may affect ant communities. Along this line, it is essential to consider that colonies of all ant species in temperate regions require multiple years to establish, grow, and reproduce (Dauber & Wolters, 2005; Seifert, 2018). Hence, in an agricultural landscape long‐term set‐aside area and durable grassland interspersion between arable fields are required to maintain ants and their functional key role as ecosystem engineers in agricultural systems.

Understanding the ecological function of a species in a particular habitat requires knowledge of species‐specific traits, their dependence on environmental factors, and ecological niches (Gagic et al., 2015). Functional traits of ants, such as colony size (number of individuals), predation on pest insects (proportion of animal‐based resources in diet), and recruitment behavior, are closely linked to the biological control services they may provide (Perović et al., 2018). Moreover, functional traits correspond to species‐specific responses of ants to habitat alteration and management intensity of agroecosystems (Ekroos et al., 2013).

This study aimed to document the development of ant community composition and functional diversity within new grassland strips of three years of age adjacent to crop fields. The results were compared to reference plots in traditionally used old grasslands, and control plots situated in the surrounding cereal crops.

At the start of their implementation, new grasslands are assumed to provide habitats primarily for common agrobiont ant species (Dauber & Wolters, 2005). We thus expected a time lag to the colonization of habitat specialists from old into new grasslands and therefore a lower complexity of the ant species community compared with surrounding old grasslands. Nevertheless, we expected ant species richness and abundance in new grasslands to be higher compared with surrounding cereal fields, as new grasslands offer more diverse ecological niches (Dauber & Wolters, 2005).

Further, we aimed to link both taxonomic composition and trait‐based composition of ants to their functional role as pest control agents in agroecosystems. Recent ecological studies highlight that functional trait diversity rather than species richness drives the delivery of key ecosystem services provided by arthropods (Gallé et al., 2019; Perović et al., 2018). Trait‐based approaches are therefore well‐suited to study the correlation between changes in community multifunctionality and delivery of agroecosystem services (Gagic et al., 2015). As functional diversity is determined by species‐specific traits, it is further important to consider which fraction out of the species community is covered by the sampling method (Gotelli et al., 2011). In this study, we investigated aboveground foraging ants, which are able to mediate key biocontrol services, and neglected leaf litter ants and those living strictly underground (Seifert, 2018; Wills & Landis, 2018). Correspondingly, we expected new grasslands to sustain the functional trait space as seen in old grasslands and to meet the same prevalence of traits, which are essential for the provision of biocontrol services.

Social insects such as ant colonies have high and continuous nutrient requirements and therefore play a key role as biocontrol agents in agroecosystems (Offenberg, 2015; Seifert, 2018). We expected new grasslands to provide a comparable amount of pest control in comparison with old grasslands and cereal fields. However, predation experiments, as a proxy for pest control, are likely influenced by microhabitat effects on foraging choices such as vegetation density and corresponding food supply. Hence, we expected a significant influence of vegetation density on measured predation rates, as we estimated a dense vegetation to reduce the dependency of predatory arthropods to feed on experimentally exposed prey (Kruess & Tscharntke, 2002).

## 2 METHODS

### 2.1 Study area

**FIGURE 1:**
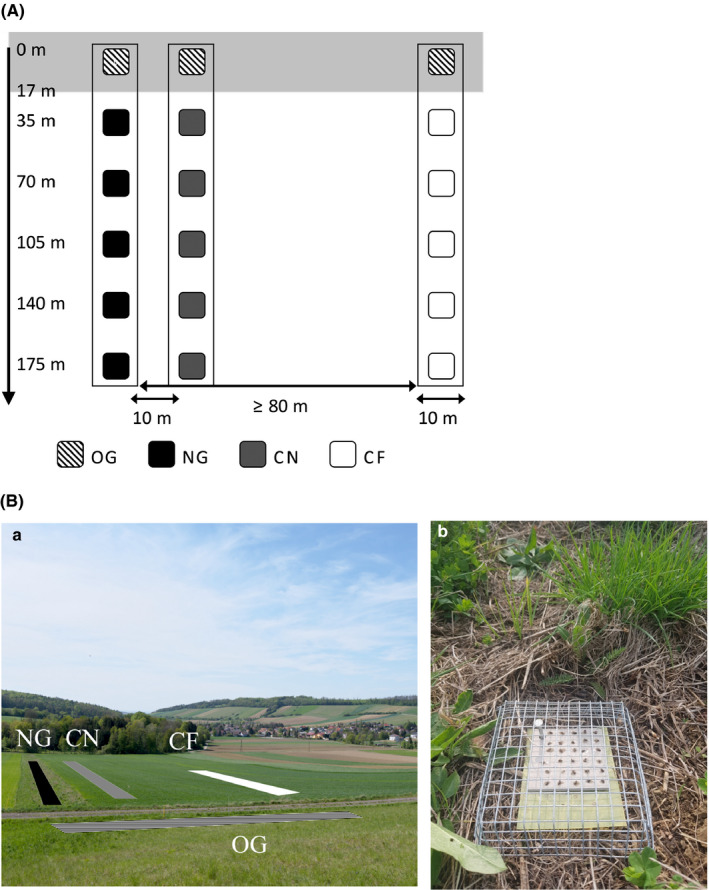
(A) Schematic sketch of ant observation sites in each study area. Each of the three transects contained six sampling plots at increasing distance to the adjacent old grassland (semi‐natural habitat remnant; gray area on top). Hatched squares (OG) = first sampling plot of each transect in old grassland (reference plots); black squares (NG) = sampling plots within new grassland; gray squares (CN) = sampling plots in adjacent cereal field near to NG; white squares (CF) = sampling plots in control cereal field far from NG. (B) Exemplary images showing the (a) study area of habitat transects (OG = old grassland; NG = new grassland; CN = cereal field near NG; CF = cereal field far from NG) in Elsbach (Lower Austria) and the (b) setup of sticky card experiment

Field experiments were performed within the framework of the Austrian research project “REGRASS” (*re‐establishing grassland strips to promote biodiversity and ecosystem services*). The study area was located near the villages Elsbach (48°15′08.3″N 16°02′56.9″E) and Ollern (48°16′02.5″N 16°05′07.9″E) in Lower Austria. The region is characterized by small‐scale but intensively managed agricultural land, along the foothills of the Wienerwald forests (mean annual air temperature and precipitation: 9.9°C, 673 mm). In this study area, five crop fields were selected, adjacent to extensively managed, semi‐natural pasture (old grasslands; OG). In each of the crop fields, three different transects directly adjacent to the old grasslands were established (see Figure 1a,b): new grasslands (NG), a transect within cereal fields ten meters next (CN) to new grasslands and within cereal fields in far (CF) distance of >80 m to new grasslands. Each transect contained six sampling plots at a regular distance of 35 m, making up 15 transects comprising 90 sampling plots in total. The first sampling plot of each transect was located in the old grasslands. Grassland ant species, as well as biocontrol potential, were investigated over a period of 2 months between 8 April and 7 June 2019.

The new grasslands had been established in August 2016 in five winter cereal fields directly adjacent to selected areas of old grassland. In order to mimic the native plant community of the old grasslands, the new grasslands were sown with a variety of seeds from 54 different plant species native to the region (with 34.1% grass species, 51.3% herbaceous plants, and 14.6% legumes). Old grasslands were extensively managed with two yearly mowing events in June and August. New grasslands were mowed once every year in August. Otherwise, there were no management interventions within new and old grasslands. The use of tillage in the cereal field transects was avoided by the farmers during the sampling period between April and June, but other forms of field management such as the use of pesticides or fertilizers were continued.

### 2.2 Data recording/sampling

Ant activity and diversity were recorded using hand collections of worker ants with fine tweezers. Hand collection is considered the most efficient method for sampling ants (Gotelli et al., 2011), and is not biased in favor of behaviorally dominant species that monopolize food resources (Andersen, 1997), which may occur when using bait traps. A total of three consecutive survey runs on each of the 90 sampling plots were performed, with 14–21 days between each run. On each sampling plot, two 1 × 1 m sized quadrants around the center were searched for foraging worker ants for 4 min each per run. Worker ants active around nests were also sampled, and the total aboveground nest activity (number of observed ants in steps of 10 individuals) was estimated. Prior to the hand sampling, local vegetation cover (0%–100% of bare soil covered) was estimated visually in a radius of 2 m around the plot center. All collected individuals were preserved in 70% ethanol and identified to species level according to Seifert (2018), using a stereomicroscope at 10‐fold magnification.

Biocontrol potential was measured using sticky card experiments with adult *Drosophila melanogaster* (Meigen) flies as baits. Predation on fruit flies is well‐suited as a general proxy for pest control, because fruit flies are available in large numbers with a body size that allows all kind of different predators to prey on them (Lys, 1995). Further, fruit flies are easily redetectable, which allows data recording. Even though highly specialized and/or parasitic predator species might not be attracted when using fruit flies as baits, the results offer general interpretation, as pest control in farmlands is foremost provided by common generalist predators (Symondson et al., 2002). We recorded each sampling plot with four consecutive survey runs (total of 720 observations/cardboards). On each sticky card, thirty flies were glued to the upper side of a 6 × 8 cm cardboard, which had a plastic underlay (to protect the card from soil moisture), and fixed to the ground with a long nail. Flies were glued to the cardboard with well‐diluted fish glue enabling ground‐dwelling predatory arthropods to remove the prey, which guarantees successful predation (Lys, 1995). Each cardboard was covered by an enclosure with an appropriate mesh size (1 × 1 cm) to prevent access of rodents and birds and allow recording of arthropod predation on fruit flies (Hulme, 1996). Two cardboards were placed on each sampling plot per survey and exposed to predatory arthropods for 2–3 hr. Afterward, predation rates (number of removed flies) and the estimated vegetation cover of the sampling plots (0%–100% of surface covered) were recorded directly in the field. We observed sticky cards for 10 min after exposure to the field, as well as during collection of the cards, to record (whenever possible) the identity of arthropod predators accessing the baits.

### 2.3 Ant traits

Life‐history traits of all ant species encountered were taken from Seifert (2017, 2018) and Arnan et al. (2017). All trait data and a detailed description of trait categories are provided in the Appendix (Tables S1 and S2). The subsequent statistical analysis determined the overall functional trait space covered by the ant communities and examined three traits in detail, which are related to the provision biocontrol services.

### 2.4 Statistical analyses

#### 2.4.1 Species diversity metrics

All analyses were conducted in R (Version 3.6.2, R Core Team, 2019). Ant species richness (ant species in transects aggregated over all three runs and pooled per habitat type) was compared across habitat types with a rarefaction–extrapolation analysis using the package “iNEXT” (Hsieh et al., 2020). For each habitat, Hill's numbers were calculated, which represent a unified family of diversity indices (Hill, 1973) and are expressed in three diversity metrics: number of species (*q* = 0), exponential Shannon's index (*q* = 1), and Simpson's index (*q* = 2). As species diversity metrics are sensitive to sample size and correspondingly to sample completeness, the expected values for each metric were displayed as functions of species coverage of the community pool. For sample sizes smaller or larger than the actual sample size in the study, estimators for each Hill number were calculated, via rarefaction or extrapolation, respectively, and curves of each biodiversity metric were presented. As suggested by Chao et al. (2014), estimators for each diversity metric were extrapolated up to a maximum of two times the real sample size and 95% confidence intervals for each diversity metric curve gained via bootstrapping.

Rarefied estimates and confidence intervals for each diversity metric were extracted from the obtained curves, corresponding to the smallest common sample size among habitats. Subsequently, a linear model using generalized least squares (GLS) was constructed for each diversity metric, in order to analyze the effect of habitat type on the respective diversity metric values separately. GLS models were constructed using the “gls” function in package “nlme” (with method = “REML”), which fits the model by maximizing the restricted log‐likelihood (Pinheiro et al., 2020).

Habitat type (OG, NG, CN, CF) served as predictor and the corresponding diversity estimates as response variable. We controlled for normality of data and distribution of residuals. In case of heteroscedasticity, we included a variance structure for the predictor via the “VarIdent” argument, which allows different variances per stratum. To access *p*‐values for pairwise tests among habitats, Tukey's post hoc tests were conducted using the package “multcomp” (Hothorn et al., 2008) and controlled for multiple comparisons. In the Appendix, we further provided tables with coefficient estimate, *t*/*z*, and *df* values for all results from models/post hoc tests. Tables were exported using the package “stargaze” (Hlavac & Marek, 2018). The same approach was applied for all subsequent GLS models.

#### 2.4.2 Nestedness and community composition

To test whether observed patterns in ant beta diversity were affected more by changes in alpha or gamma diversity, nestedness measurements “NODF” and matrix temperature “T” were calculated using the package “vegan” (Oksanen et al., 2018). Gained results were verified against a constraint null model type “r00.” Nestedness measurements and null model type were chosen based on recommendations in a comprehensive literature review (Ulrich et al., 2009).

To study habitat effects on ant species composition across transects, a constrained ordination analysis was performed. As the observed abundance of foraging workers was influenced by, for example, life cycle stage of ant colonies (Seifert, 2018), the analysis was based on a pseudo‐abundance matrix, which refers to the presence of the respective species on the number of runs (0–3) on each plot (pooled per transect). A dummy species with an abundance of one in all samples was added, to deal with low numbers of species per transect (Clarke et al., 2006). Based on this dataset, a Sørensen dissimilarity matrix was created using the function “vegdist” (with method = “bray”’) in package “vegan” (Oksanen et al., 2018). Subsequently, a canonical analysis of principal coordinates with two axes on the Sørensen dissimilarity matrix was performed using function “capscale,” with habitat type as a constraint variable.

To test whether differences in ant community composition were influenced by habitat type, a PERMANOVA was conducted via “adonis” function with 999 permutations, whereby the gained Sørensen dissimilarity matrix of transects served as the input matrix. The variability of ant community composition among habitat types was analyzed via homogeneity of dispersion tests (function “betadisper”) and subsequent ANOVAs, in order to compare the mean distance to centroid of the distinct ant communities. Significant ANOVA results (*p* < .05) were followed by Tukey's post hoc test for pairwise comparisons among habitat types. The same approach was applied for the analysis of the functional trait space (see Section 2.4.3).

#### 2.4.3 Functional trait space

A principal component analysis of the species trait data was performed using the package “FactoMineR” (Lê et al., 2008), and the first two principal coordinates of each ant species were plotted in a two‐dimensional diagram. In order to display the functional trait space covered by ants in the different study habitats, a convex hull (polygon) was drawn around the respective species communities. Differences in distribution and variability of the functional trait space were tested for significance among habitat types with a PERMANOVA and homogeneity of dispersion tests based on an Euclidean distance matrix of the scaled and centered species trait data.

#### 2.4.4 Prevalence of biocontrol‐related functional traits

Community‐weighted mean (CWM) values of selected ant species traits were calculated using the “FD” package (Laliberté et al., 2014). The calculated CWM values refer to the average of species trait values at each sampling transect weighed by the relative species abundance (Ricotta & Moretti, 2011). The analysis was based on the pseudo‐abundance matrix as described above. Cereal field habitats CF and CN were excluded from the analysis, as more than half of CF and CN transects showed insufficient number of species (≤2 ant species per transect) for reliable CWM calculation (Laliberté et al., 2014). Hence, only grassland habitats OG and NG were tested for differences in the prevalence of biocontrol‐related traits.

Three traits were chosen, corresponding to their relevance for the provisioning of pest control services: *proportion of animal‐based resources* in ant diet (*Zoopha*; food resources acquired via predation or scavenging, see Table S1), *recruitment behavior* of workers (*FS*; foraging strategy), and *colony size* (*CS*; number of individuals). Values for all three traits were scaled and centered (*z*‐transformed) prior to CWM calculation and gained CWM values controlled for normality of data distribution before further analysis.

Subsequently, a linear model using generalized least squares (GLS) was constructed for each trait, in order to analyze the effect of habitat type (predictor) on the respective CWM values (response) separately. GLS models were constructed using the “gls” function in package “nlme” (with method = “REML”). To model the potential spatial dependency of transects within a study area (Elsbach, Ollern), we included an autocorrelation function to the GLS models via the “corAR1” argument. *p*‐values for pairwise comparisons were computed via Tukey's post hoc tests (see Section 2.4.1 for a detailed description of the GLS and post hoc test approach).

#### 2.4.5 Predation rates and aboveground ant activity

To investigate the effect of habitat type and mean vegetation cover rate (0%–100% of sampling plot surface covered) on predation intensity on sticky cards, a predation rate (0–1) was calculated based on the number of eaten flies per sampling plot summed across all four survey runs (*n* per 240 flies in total; 30 flies × 2 cards per plot × 4 runs). Predation rates and mean vegetation cover rates were logit‐transformed to improve normality for ratios between 0 and 1 (Warton & Hui, 2011). Subsequently, a linear model using generalized least squares (GLS) was constructed, where logit‐transformed predation rate served as response variable and habitat type as well as logit‐transformed vegetation cover rate as predictor variables. Further, we included an autocorrelation function via the “corAR1” argument to model the potential spatial dependency corresponding to the study area (Elsbach/Ollern). The best model was chosen based on comparisons of fixed effect structures and different autocorrelations using ANOVAs and AIC scores. *p*‐values for pairwise comparisons were computed via Tukey's post hoc tests (see Section 2.4.1 for a detailed description of the GLS and post hoc test approach).

To study the effect of habitat type on aboveground ant activity, the number of observed worker ants per sampling plot was summed across all three survey runs and transformed with Tukey's ladder of powers, in order to attain normally distributed values, using the package “rcompanion” (Magnificio, 2019). Subsequently, a linear model using generalized least squares (GLS) was constructed, where Tukey's transformed number of workers served as response variable and habitat type as predictor variable. Further, we included an autocorrelation function as described above. The best model was chosen based on comparisons of fixed effect structures and different autocorrelations using ANOVAs and AIC scores. *p*‐values for pairwise comparisons were computed via Tukey's post hoc tests (see Section 2.4.1 for a detailed description of the GLS and post hoc test approach). Further, the correlation of predation rate on sticky cards and Tukey's transformed aboveground ant activity was tested with a GLM and the calculation of *R* ^2^ value.

## 3 RESULTS

### 3.1 Ant species richness

In total, 11 ant species were collected in the four habitats over all 90 sampling plots (see Table S3 for a detailed abundance description). A cumulative number of 8 species were found in each grassland habitat (OG and NG), whereby only 3 and 2 species were recorded in cereal fields CN and CF, respectively.

Three species occurred only on one sampling plot, *Formica rufa* (one individual found in new grassland)*, and Lasius fuliginosus* and *Serviformica cunicularia* (each one individual found in old grassland), and were excluded from the analysis as they likely present stray individuals. *Lasius niger* was the most frequent and widespread species, occurring on 82 plots and in all four habitats. The species complex *Lasius alienus* agg. occurred on 7 plots and was unique for old grasslands.

Rarefied estimates for species richness (Figure 1a, calculated for the smallest common sample size of CN = 42 individuals; Tables S4 and S5a) were not significantly different between grassland habitats (*p*‐value = .993), but significantly higher in OG compared with cereal field habitats CF and CN (*p*‐value <.05). New grasslands showed a strong trend for higher species richness compared with CF (*p*‐value = .054), but no significant difference compared with CN (*p*‐value = .360).

Rarefied estimates for Shannon's diversity index (Figure 1b; Tables S4 and S5b) showed a trend for higher values in grassland habitat OG compared with NG (*p*‐value = .08) and were significantly higher in OG compared with both cereal field habitats (*p*‐value <.05). New grasslands showed a trend for higher values regarding Shannon's diversity compared with CF (*p*‐value = .089), but no significant difference compared with CN (*p*‐value = .862).

Rarefied estimates for Simpson's diversity index (Figure 1c; Tables S4 and S5c) were significantly highest for grassland habitat OG compared with all other habitats (*p*‐value <.05). Grassland habitat NG and cereal fields CF and CN did not significantly differ among each other regarding estimated values for Simpson's diversity (*p*‐value >.1).

### 3.2 Nestedness and composition of ant communities

The nestedness analysis revealed no significant differences between the ant assemblages of the four habitats and a maximally nested null model. Thus, the results indicated a high degree of nestedness in the overall ant community (NODF sites = 58.82, matrix temperature *T* = 26.01; proportional row and column totals of the observed statistic based on simulations: *z* = 1.24 and *z* = 0.35, respectively; *p* = .63).

CAP ordination revealed that ant community composition across transects was significantly affected by habitat type (*p* = .001, see Table S6a for PERMANOVA results). Data points referring to ant community composition of old grasslands (light green squares, see Figure 3) were (except of one) clearly separated from other habitats, according to their position along the first ordination axis. Contrary, data points referring to ant community composition at new grasslands (dark green triangles) clustered together with data points from cereal field habitats (purple circles and blue diamonds). Homogeneity of dispersion tests showed significant differences (*p*‐value = .043, see Table S6b) in variability of ant community composition among habitat types, which referred to significantly higher variability in habitat OG compared with CF (*p*‐value = .042, see Table S6c).

### 3.3 Principal component analysis of functional trait space

Principal component analysis showed that the distribution and the variability of the functional trait space covered by ant species communities were not significantly affected by habitat type (PERMANOVA: *p* = .994, see Table S7a; homogeneity of dispersion test: *p*‐value = .613, see Table S7b). The trait spaces covered by the ant communities of cereal fields (purple dashed polygon, see Figure 4) and new grasslands (dark green dashed polygon) showed more or less the same distribution. Both were determined by merely three species: *Lasius niger*, *Serviformica rufibarbis,* and *Myrmica rugulosa*/*Myrmica schencki*. The trait space occupied in old grasslands (light green dashed polygon) was slightly smaller and determined by the species *Lasius niger*, *Serviformica rufibarbis*, *Myrmica schencki,* and *Myrmica scabrinodis*.

### 3.4 Community‐weighted means of traits related to biocontrol services

The comparison of community‐weighted means (CWM) focused on three species traits related to biocontrol potential of the ants and the old and new grassland habitats. Results for both cereal field habitats are not shown, due to insufficient species richness for CWM calculation. CWM values of the proportion of animal‐based resources in ant diet (Figure 5a) were not significantly different between old and new grasslands (*p* = .837, see Tables S8a and S8b), and analogous results were found for the CWM values of food recruitment strategy (Figure 5b, *p* = .95) and ant colony size (Figure 5c, *p* = .953).

### 3.5 Predation intensity on sticky cards and aboveground ant activity

Predation rate on fruit flies glued on sticky cards (see Figure 6a) was highest for sampling plots in cereal fields far from new grasslands and lowest for new grasslands, but showed no significant differences among the four tested habitats (*p* ≥ .59 for all comparisons; see Tables S9 and S10). These results relate to a negative correlation of predation rate and mean vegetation cover, which was higher in grassland habitats OG and NG compared with cereal field habitats CF and CN (see Table S9). During recollection of cardboards, which corresponds to a total of ~6 hr of direct observations in the field, predators removing the fruit flies were observed on 274 out of 720 cardboards (38.06%; see Table S12). Ants contributed 50.36% to the total number of observations on cardboards and were hence the most common predators observed, followed by carabid beetles (21.9%) and wasps and/or flies (12.04%).

Aboveground ant activity (number of observed workers, see Figure 6b) was significantly highest on sampling plots in old grasslands (*p* < .001, see Tables S11a and S11b). Moreover, ant activity was significantly increased on sampling plots in new grasslands compared with cereal field habitats CN and CF (*p* < .01), which did not significantly differ between each other (*p* = .980).

There was no significant correlation between aboveground ant activity and predation intensity on sticky cards (*R* ^2^ ≤ 0.001, *p* = .990).

## 4 DISCUSSION

### 4.1 Species richness and functional diversity

**FIGURE 2:**
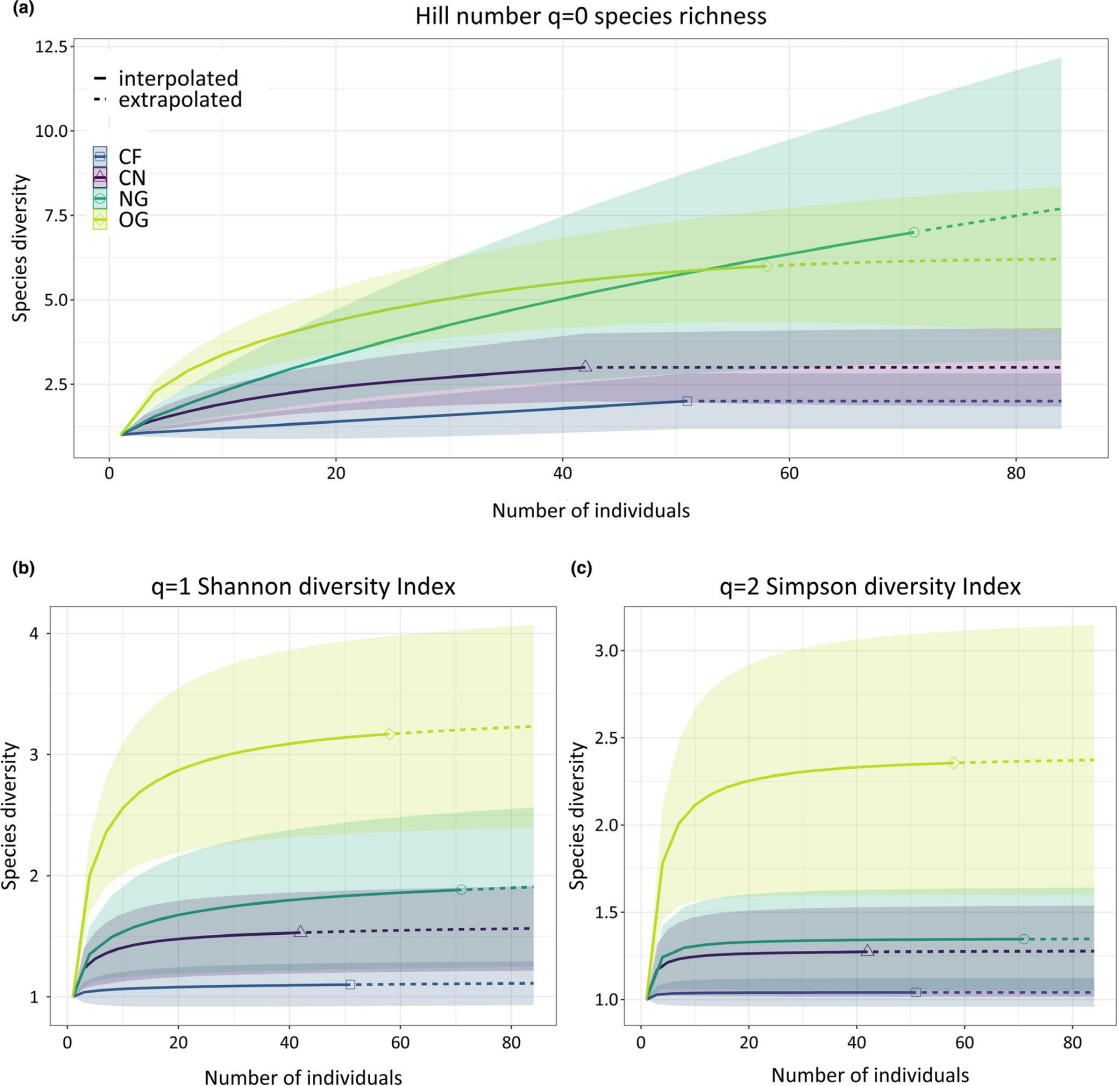
(a–c) Integrated diversity metric curves (gained from rarefaction/extrapolation) displayed as a function of sample size for each habitat (OG = old grassland; NG = new grassland; CN = cereal field near NG; CF = cereal field far from NG). Three diversity metric curves (Hill numbers *q*) are shown: the (a) species richness (*q* = 0), (b) Shannon's diversity index (*q* = 1), and (c) inverse Simpson's index (*q* = 2). Colors and shapes indicate the respective habitat identity. Solid curves denote interpolation, and dashed lines, extrapolation estimates

Our results demonstrate the potential of new grasslands to promote ant species richness in agricultural landscapes, but only if preserved over long periods of time. Rarefied estimates for species richness were comparable between old and new grasslands and significantly higher in old grasslands compared with surrounding cereal fields (Figure 2a, Tables S4 and S5a). New grasslands showed a strong trend for higher species richness compared with cereal fields.

However, even though species richness and abundance had already increased to higher levels after three years of establishment, the new grasslands were still less diverse and lacked rare ant species present in old grasslands. The latter was expressed in lower rarefied estimates for Shannon's diversity and significantly lower estimates for inverse Simpson's index in new grasslands compared with old grasslands (Figure 2b,c, Tables S5ab and S5c). Furthermore, new grasslands showed similar estimates for the latter diversity metrics compared with cereal fields.

**FIGURE 3:**
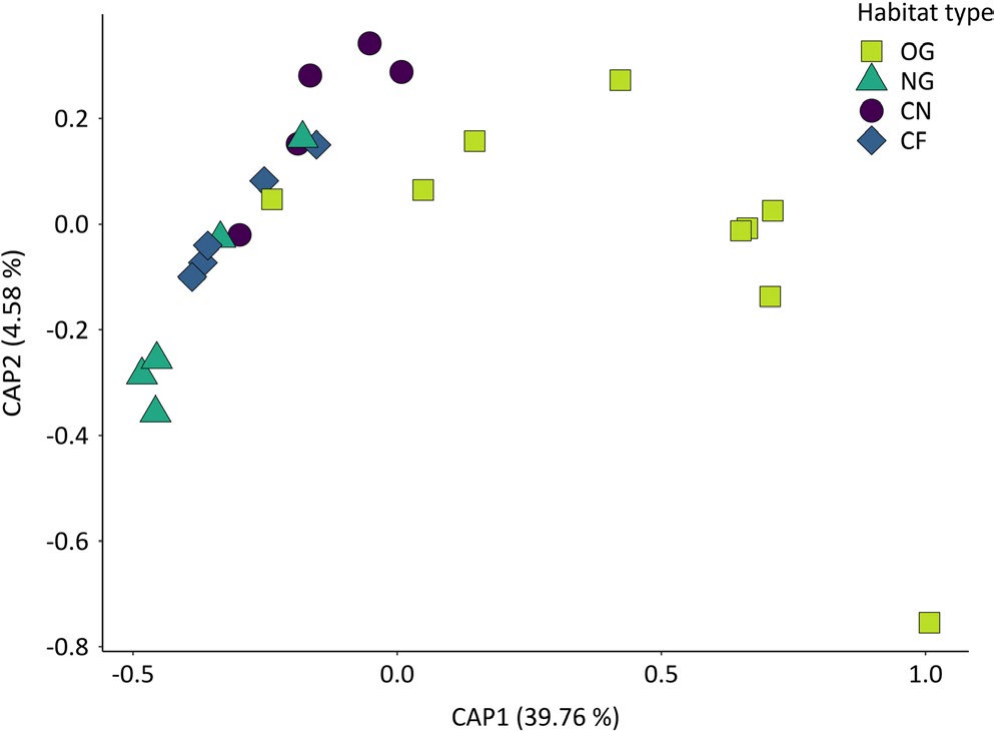
Ant community composition of habitats. Ordination plot of canonical analysis of principal coordinates (CAP) showing the influence of the constraint variable habitat type (OG = old grassland; NG = new grassland; CN = cereal field near NG; CF = cereal field far from NG; differently colored and shaped symbols, respectively) on ant species composition. Each symbol indicates ant species composition of one sampled transect. Symbols have been slightly shifted to reduce overlap. Values on CAP axes refer to the percentage of explained variance (eigenvalues)

**FIGURE 4:**
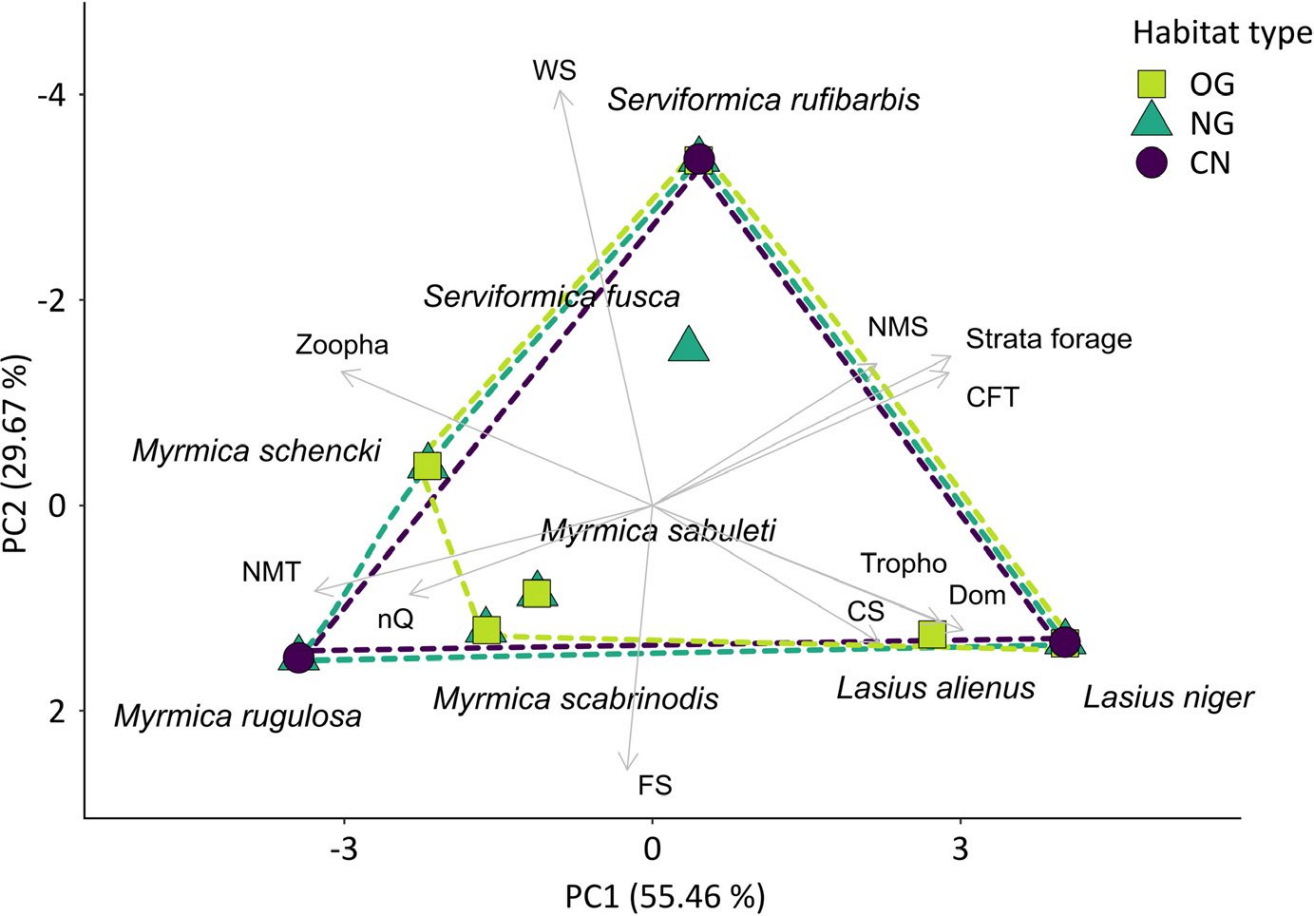
Ordination plot showing the trait space covered by ant species occurring in three of the four habitat types (OG = old grassland; NG = new grassland; CN = cereal field near NG; differently colored and shaped symbols and dashed lines, respectively). CF samples (cereal field far from NG) are not shown due to low cumulative species richness. A principal component analysis (PCA) was conducted based on a species trait matrix. Each symbol indicates the position of one species occurring in the respective habitat. Arrows indicate the correlation of the respective traits with the species position in reduced ordination space. Used traits are as follows: Vertical strata species is most likely to be found foraging (Strata_forage), percentage of animal diet among total food intake (Zoopha), percentage of trophobiosis‐based diet of total food intake (Tropho), worker body length in mm (WS), colony size log‐transformed (CS), behavioral dominance (Dom), number of queens per nest (nQ), colony foundation type (CFT), recruitment behavior of workers (FS), percentage of microhabitats in soil and/or under stones contributing to total nest space (NMS), and percentage of microhabitats in upper root mat contributing to total nest space (NMT). Values on PCA axes refer to the percentage of explained variance (eigenvalues)

**FIGURE 5:**
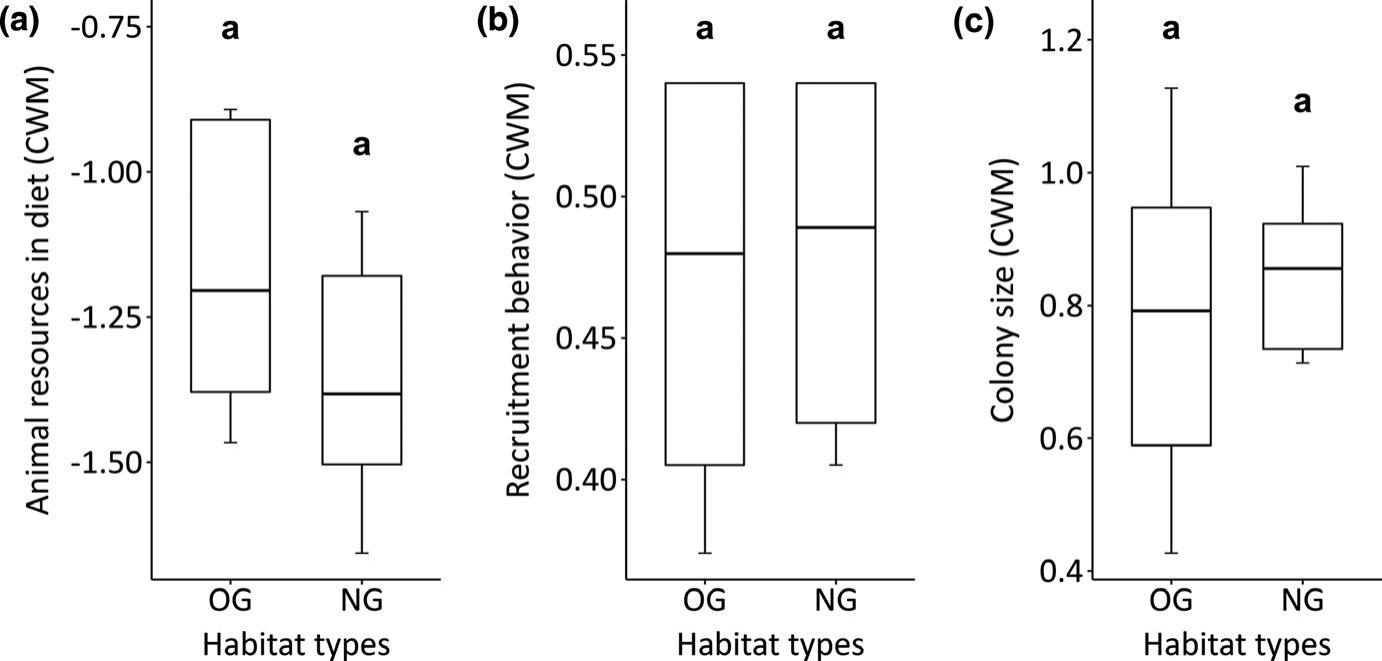
(a–c) Biocontrol‐related ant species traits in old and new grasslands. For each habitat (OG = old grassland; NG = new grassland), the CWM values of the (a) proportion of animal‐based resources in ant diet, the (b) recruitment strategy (workers forage individually (0), workers guide a low number of nest mates to a previously discovered food source (0.5), workers can trigger mass recruitment (1)), and the (c) colony size are shown. Traits have been scaled and centered prior to CWM calculation, and *y*‐axes hence show transformed values. Boxes represent CIs, lines represent means, and whiskers represent ranges. Letters indicate results of Tukey's post hoc test of fixed factor levels (GLS analysis). Different letters denote significant (*p* < .05) differences between habitats. Results for habitats CN and CF are not shown, as the respective species richness was insufficient for CWM calculation

The nestedness analysis provided further evidence that the observed patterns in beta diversity were affected by changes to alpha diversity, meaning that new grasslands and cereal field habitats comprised a depleted species selection out of the larger species pool present in old grasslands (see Results 3.2). These results were supported by ordination analysis (Figure 3, Table S6), showing that the ant community composition of new grassland transects was mostly shaped by ubiquitous agrobiont species, such as *L. niger* and a few *Myrmica* species. Both of them are known to be resistant to anthropogenic disturbance (Seifert, 2018) and also inhabited cereal fields. In line with previous studies (Dauber & Wolters, 2005), we found that after three years new grasslands were still in earlier stages of ant community succession and lacked habitat specialists such as *L. alienus* agg., presenting a characteristic species of extensively managed grasslands (Seifert, 2018). Colonies of these species require a constant supply of food resources and take several years to establish, grow, and reproduce (Dauber & Wolters, 2005; Seifert, 2018). Contrary to the less complex ant community composition found in new grasslands, principal component analysis showed that new grasslands were able to meet the same functional trait diversity as seen in old grasslands. The functional trait space in new grasslands was determined by three common agrobiont species (*L. niger*, *S. rufibarbis*, and *M. rugulosa*), which were also present within cereal field habitats (Figure 4). However, this fraction out of the local species community already provided three functional traits essential for biocontrol services, namely a predatory diet, the ability of workers to organize mass recruitment of nest mates, and large colony sizes (Figure 5a–c, Tables S8a and S8b).

The aim of this study was to link taxonomic and trait‐based composition of ants to their functional role as mediators of pest control services in agroecosystems. Our results indicate that even though new grasslands do not possess the same faunal complexity as seen in old grasslands, they are able to increase ant species diversity and to sustain biocontrol essential functional traits in farmlands (Dauber & Wolters, 2005). Nevertheless, our sampling effort was probably too low to detect rare species, as indicated by the three‐singleton workers of ant species that form particularly large long‐lived colonies. According to Seifert (2018) and comparable studies, 30–40 different ant species can be expected in extensively managed grasslands habitats in Central Europe, such as the old grasslands in our study (Heuss et al., 2019). Several of these species were not assessed, also due to our restriction to sample only aboveground foraging ants, but serve other key ecological functions such as seed dispersal, controlling the abundance of honeydew‐producing insects and regulating soil parameters (Wills & Landis, 2018).

In account of the loss of biodiversity and associated ecosystem services in agricultural landscapes (Cardoso et al., 2020), we want to emphasize the multifunctional role of ants as ecosystem engineers. New grasslands need to be established for longer periods of time to allow colonization of ecologically sensitive species and to substantially enhance ant species diversity (Dahms et al., 2010). Comprehensive species immigration from the regional species pool is indispensable for sustaining all key biological functions provided by ants in agroecosystems (Wills & Landis, 2018). We conclude that short‐term measures need to be complemented by the conservation of durable semi‐natural grassland areas to extend the range of suitable foraging and nesting sites for ants (Armbrecht et al., 2004) and further the contribution that ants and other arthropods may provide to agroecosystem functioning in their surroundings.

### 4.2 Predation experiments

**FIGURE 6:**
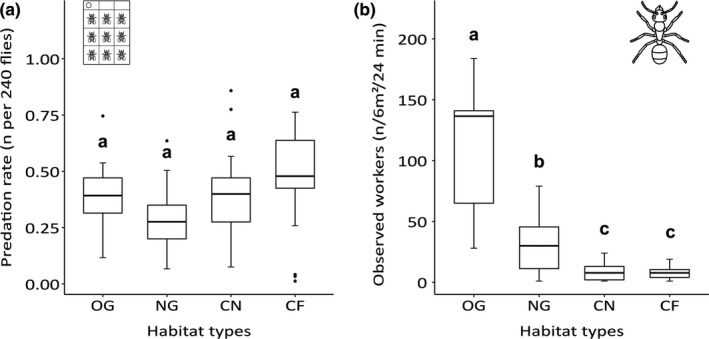
(a and b) Predation rate on sticky cards and aboveground ant activity. For each habitat (OG = old grassland; NG = new grassland; CN = cereal field near NG; CF = cereal field far from NG), the (a) predation rate on sticky cards per plot summed across all 4 runs (*n* per 240 flies) and the (b) ant activity as the number of aboveground foraging ants per sampling plot summed across all 3 runs (observed workers per 6 m² and 24 min in total) are shown. Boxes represent CIs, lines represent means, and whiskers represent ranges. Letters indicate results of Tukey's post hoc test of fixed factor levels (GLS analysis). Different letters denote significant (*p* < .05) differences between habitats

Grasslands and cereal fields showed no difference in predation rates, but new grasslands promoted ant activity compared with levels seen in cereal fields (Figure 6a,b, Tables S9, S10, S11a, and S11b). Although the spatial scale of our experiments was comparably small, the results suggest that new grasslands embedded in agricultural landscapes can promote biological control. Our results showed that bait predation was generally lower on sites with higher vegetation density, such as new and old grasslands, while habitat type played only a minor role (see Tables S9 and S10). This supports our assumption that predation experiments are highly influenced by microhabitat effects on foraging choices, meaning that the attractiveness of bait flies is generally lower on sites with higher vegetation density and corresponding greater food supply (Kruess & Tscharntke, 2002).

For the management of pest control, it is important to consider that biocontrol services are not provided solely by ants, but by a diverse assemblage of ground‐dwelling arthropod predators in the agricultural matrix (Meyer et al., 2019). Carabids, spiders, wasps, and predatory flies were also able to access the baits on the cardboards and their contribution, which was not assessed in this study, likely impeded a correlation of predation rate and ant activity.

We observed sticky cards for 10 min after exposure to the field, as well as during recollection of the cards to get some additional information on predator activity while keeping observation disturbance levels low. A total of approx. six observation hours showed that ants were the most common group of predatory arthropods accessing fruit fly baits, next to carabid beetles and wasps and/or flies (Table S12). Even though these numbers do not show the true contribution of each predatory group to predation, as cardboards were not observed for the whole exposure time in the field, they highlight the potential of ants as biocontrol agents in agroecosystems. Future studies may resolve the issue of multitrophic interactions with the implementation of finer mesh sizes covering the sticky cards to exclude predators such as large carabids, wasps, flies, and spiders. Further, predation experiments should be conducted on a coarser scale, in order to better highlight differences regarding pest control services in complex structured farmlands.

Nevertheless, the results of the predation experiment are relevant because new grasslands foster the abundance and species richness of ants, which account for a significant part of the biomass of predatory arthropods in certain agricultural landscapes (Wills & Landis, 2018). In conclusion, new grasslands promote the provision of biocontrol with increasing age and due to growing arthropod populations, including ant colonies.

### 4.3 Synthesis and applications

Our findings not only show that new grasslands can increase ant species richness, abundance, and potentially also pest control in agroecosystems, but also indicate that it takes longer than three years to regain biodiversity levels comparable to those in old semi‐natural grasslands. To counteract the loss of biodiversity and related functions, agricultural management should consider key strategies for ecological enhancement (Bommarco et al., 2013; Perović et al., 2018). Service providing arthropods can be promoted through the enhancement of floral nectar resource availability, which promotes longevity and fertility of biocontrol agents (Perović et al., 2018). Other strategies include landscape‐level diversification, diversified crop rotations, and the reduction in harmful measures, such as pesticide application and frequent ploughing (Bommarco et al., 2013).

Our findings illustrate that new grasslands should be integrated into a long‐term management strategy for the promotion and resilience of yield‐enhancing ecosystem services provided by ants. Long‐term establishment of new grasslands is required, because a turnover back into crop fields inevitably destroys initiated ant colonies, disrupts ant community succession, and dramatically reduces arthropod populations that deliver key biocontrol services (Ganser et al., 2019). Moreover, long‐term establishment is paramount to promote not only ubiquitous ant species in their abundance but also habitat specialists with longer colonization times (Dauber & Wolters, 2005). Ecological enhancement strategies should acknowledge that only a broad diversity of functional insurance species can guarantee the resilience of biological control services in European agroecosystems (Tscharntke et al., 2005). Our findings suggest that new grasslands represent a promising measure for enhancing agricultural landscapes and should be contemplated by European policy and agricultural decision makers. However, effective agri‐environment schemes need to consider that long‐term set‐aside areas and durable grassland interspersion are required to allow comprehensive immigration of ant species into habitats that support agricultural biodiversity and functionality.

## CONFLICT OF INTEREST

The authors have no conflict of interest to declare.

## AUTHOR CONTRIBUTIONS

**Victor Sebastian Scharnhorst:** Conceptualization (supporting); data curation (lead); formal analysis (lead); investigation (equal); methodology (equal); visualization (lead); writing‐original draft (lead); writing‐review & editing (equal). **Konrad Fiedler:** Conceptualization (supporting); data curation (supporting); formal analysis (supporting); investigation (supporting); methodology (supporting); supervision (supporting); validation (supporting); visualization (equal); writing‐original draft (equal); writing‐review & editing (equal). **Thomas Frank:** Conceptualization (supporting); formal analysis (supporting); funding acquisition (lead); investigation (supporting); methodology (supporting); project administration (supporting); resources (lead); supervision (supporting); validation (equal); visualization (equal); writing‐original draft (equal); writing‐review & editing (equal). **Dietmar Moser:** Conceptualization (supporting); data curation (supporting); formal analysis (supporting); funding acquisition (lead); investigation (supporting); methodology (supporting); project administration (supporting); resources (lead); supervision (supporting); validation (equal); visualization (equal); writing‐original draft (equal); writing‐review & editing (equal). **Dominik Rabl:** Data curation (equal); resources (equal); writing‐review & editing (equal). **Manuela Brandl:** Project administration (equal); resources (equal); writing‐review & editing (equal). **Raja Imran Hussain:** Investigation (supporting); methodology (supporting); project administration (supporting); writing‐review & editing (equal). **Ronnie Walcher:** Investigation (supporting); methodology (supporting); writing‐review & editing (equal). **Bea Maas:** Conceptualization (lead); data curation (equal); formal analysis (equal); investigation (lead); methodology (lead); project administration (lead); supervision (lead); validation (equal); visualization (equal); writing‐original draft (equal); writing‐review & editing (equal).

### OPEN RESEARCH BADGES

This article has earned an Open Data Badge for making publicly available the digitally‐shareable data necessary to reproduce the reported results. The data is available at https://doi.org/10.5061/dryad.z08kprrcm.

## Supporting information

Supplementary Material

## Data Availability

Most of the data supporting the findings of this study at the journal level are available within the paper (and its Supporting Information files). The entire datasets that support the findings of this study are available from Dryad (https://doi.org/10.5061/dryad.z08kprrcm). Extended data are available on request from the authors.
