# Supplementary material for: Ant community composition and functional traits in new grassland strips within agricultural landscapes
Source: Ecol Evol. 2021 May 25;11(12):8319–31. doi: 10.1002/ece3.7662 (PMC8216914; doi:10.1002/ece3.7662)
Supplement: Supplementary file 1 — Supplementary Material [file ECE3-11-8319-s001.docx]

# Appendix (Scharnhorst et al.)

## **S1 – Ant species and traits**

*Table S1. Traits of grassland ant species used for this study. Abbreviations:* ***Zoopha*** *= assumed percentage of animal diet among total food intake;* ***Tropho*** *= assumed percentage of trophobiosis based diet of total food intake;* ***WS*** *= worker size (body length in mm), measures were taken during the REGRASS project by Dominik Rabl;* ***CS*** *=colony size (ln transformed);* ***Dom*** *= behavioral dominance;* ***nQ*** *= number of queens per nest;* ***CFT****= colony foundation type;* ***Strata forage*** *= vertical strata where species is most likely to be found foraging (calculated as the sum of the probability of worker ants to forage in different vertical strata, where the value for each stratum is multiplied by a specific factor, based on predictions of Seifert 2017); positive values indicate higher probability of species to be found foraging above ground; negative values: foraging under the surface;* ***FS*** *= recruitment behaviour (foraging strategy);* ***NMS*** *= assumed percentage of microhabitats in soil and/or under stones contributing to total nest space;* ***NMT*** *= assumed percentage of microhabitats in upper root felt and lowest epigaean parts of grasses contributing to total nest space.*

| ***Species*** | ***Functional traits*** | | | | | | | | | | |
| --- | --- | --- | --- | --- | --- | --- | --- | --- | --- | --- | --- |
|  | **Zoopha** | **Tropho** | **WS** | **Dom** | **CS** | **nQ** | **CFT** | **Strata forage** | **FS** | **NMS** | **NMT** |
| *Lasius alienus* agg. | 0.39 | 0.37 | 3.693 | 1 | 9.47 | 0 | 1 | 0.76 | 1 | 0.82 | 0.09 |
| *Lasius niger* | 0.34 | 0.56 | 3.734 | 1 | 9.21 | 0 | 1 | 1.05 | 1 | 0.83 | 0.04 |
| *Serviformica fusca* | 0.5 | 0.35 | 5.895 | 0 | 9.1 | 1 | 1 | 1.08 | 0.5 | 0.59 | 0.07 |
| *Serviformica rufibarbis* | 0.59 | 0.31 | 6.25 | 0 | 6.91 | 0 | 1 | 1.04 | 0.5 | 0.87 | 0.09 |
| *Myrmica rugulosa* | 0.64 | 0.25 | 4.359 | 0 | 7.6 | 1 | 0.5 | 0.13 | 1 | 0.48 | 0.24 |
| *Myrmica sabuleti* | 0.51 | 0.37 | 4.793 | 0 | 8.01 | 1 | 0.5 | 0.54 | 1 | 0.74 | 0.15 |
| *Myrmica scabrinodis* | 0.51 | 0.4 | 4.647 | 0 | 7.31 | 0.5 | 0.5 | 0.34 | 1 | 0.59 | 0.2 |
| *Myrmica schencki* | 0.58 | 0.27 | 5.426 | 0 | 5.87 | 0.5 | 0.5 | 0.29 | 1 | 0.75 | 0.15 |

**S2 – Description of ant species traits**

*Table S2. Description of grassland ant species traits used for this study. Trait information marked with * was taken (partly) from Seifert (2007, 2017), scorings marked with + were taken from Arnan et al. (2017) and those marked with ° were taken from Heuss et al. (2019).*

| **Trait** | **Data type** | **Character states** |
| --- | --- | --- |
| CS+* | Continuous | Mean colony size (log transformed) |
| WS* | Continuous | Worker body size [mm] |
| Zoopha* and Tropho* | Continuous | Assumed relative percentage of animal- or trophobiosis-based resources in diet. |
| Dom+* | Binary | (0) Subordinate; (1) Dominant |
| nQ+* | Ordinal | (0) Monogyny; (0.5) Monogyny or polygyny; (1) Polygyny |
| CFT+* | Ordinal | (0) Dependent colony founding; (0.5) Dependent and independent colony founding; (1) Independent colony founding |
| Strata_forage° | Continuous | Positive values for higher probability of species found foraging above ground, negative values for foraging under the surface. |
| FS+* | Ordinal | (0) Workers forage and collect food individually; (0.5) workers communicate and guide a low number of nestmates to a previously discovered food source; (1) workers follow “anonymous” chemical signals provided by other nestmates and can organize mass-recruitment to exploit a food source |
| NMS* and NMT* | Continuous | Assumed relative percentage of different strata contributing to the true nest space microhabitat: in soil and/or under stones (NMS), in upper root felt and lowest epigeic parts of grasses (NMT) |

**S3 – Ant species abundance per habitat**

*Table S3. Abundance description of ant species found in the study area in the four sampled habitats (OG = old grassland; NG = new grassland; CN = cereal field near NG; CF = cereal field far from NG). For each species the occurrence on n out of 90 plots in total and for each habitat is shown.*

| **Species** | **Total n**  **(all habitats)** | **n (CF)** | **n (CN)** | **n(NG)** | **n(OG)** |
| --- | --- | --- | --- | --- | --- |
|  |  |  |  |  |  |
| *Formica rufa* | 1 | 0 | 0 | 1 | 0 |
| *Lasius fuliginosus* | 1 | 0 | 0 | 0 | 1 |
| *Lasius alienus* agg. | 7 | 0 | 0 | 0 | 7 |
| *Lasius niger* | 82 | 24 | 21 | 24 | 13 |
| *Serviformica cunicularia* | 1 | 0 | 0 | 0 | 1 |
| *Serviformica fusca* | 2 | 1 | 0 | 1 | 0 |
| *Serviformica rufibarbis* | 4 | 0 | 1 | 1 | 2 |
| *Myrmica rugulosa* | 7 | 0 | 3 | 4 | 0 |
| *Myrmica sabuleti* | 3 | 0 | 0 | 2 | 1 |
| *Myrmica scabrinodis* | 3 | 0 | 0 | 1 | 2 |
| *Myrmica schencki* | 2 | 0 | 0 | 1 | 1 |
| No. species (total) | 11 | 2 | 3 | 8 | 8 |
| No. species (unique) | 4 | 0 | 0 | 1 | 3 |

| **S4 – Rarefied diversity analysis: GLS results**  *Table S4: GLS regression results for the rarefied diversity estimates (Hill’s numbers) corresponding to the smallest common sampling size across habitats (CF = 42). For each Hill’s number the coefficient estimate, standard error (in brackets) and t-value corresponding to each habitat type (OG = old grassland; NG = new grassland; CN = cereal field near NG; CF = cereal field far from NG) is shown.* | | | |
| --- | --- | --- | --- |
|  | | | |
| *Predictor variable:* | *Response variable:* | | |
|  |  | | |
| Habitat type | Hill's numbers q | | |
|  | q = 0 | q = 1 | q = 2 |
| CF | 1.824 | 1.097^***^ | 1.040^***^ |
|  | (0.500) | (0.091) | (0.042) |
|  | t = 3.650 | t = 12.102 | t = 24.838 |
| CN | 1.176 | 0.432^*^ | 0.233 |
|  | (0.741) | (0.233) | (0.153) |
|  | t = 1.587 | t = 1.857 | t = 1.520 |
| NG | 3.355^**^ | 0.708^**^ | 0.302^**^ |
|  | (1.334) | (0.308) | (0.151) |
|  | t = 2.516 | t = 2.298 | t = 2.004 |
| OG | 3.754^***^ | 2.004^***^ | 1.295^***^ |
|  | (0.950) | (0.478) | (0.333) |
|  | t = 3.953 | t = 4.195 | t = 3.894 |
|  | | | |
| Observations (df) | 12 | 12 | 12 |
| Log Likelihood | -15.348 | -6.101 | -1.684 |
| Akaike Inf. Crit. | 46.695 | 28.201 | 19.368 |
| Bayesian Inf. Crit. | 47.331 | 28.837 | 20.003 |
|  | | | |
| *Note:* ^*^p^**^p^***^p<0.01 | | | |

**S5 – Rarefied diversity analyses: Tukey’s post hoc tests**

*Table S5a: Tukey’s post hoc test results of GLS regression for the rarefied diversity estimates (Hill’s numbers) corresponding to* ***q = 0 (species richness)****. For each pair-wise comparison (OG = old grassland; NG = new grassland; CN = cereal field near NG; CF = cereal field far from NG) the coefficient estimate, standard error, z-value and corresponding p-value is shown.*

| Comparison | Estimate | Std. Error | Z value | p-value |
| --- | --- | --- | --- | --- |
| CN-CF | 11.763 | 0.7411 | 1.587 | 0.3731 |
| NG-CF | 33.547 | 13.336 | 2.516 | 0.0539 |
| OG-CF | 37.543 | 0.9498 | 3.953 | <0.001 |
| NG-CN | 21.783 | 13.521 | 1.611 | 0.3596 |
| OG-CN | 25.780 | 0.9757 | 2.642 | 0.0384 |
| OG-NG | 0.3997 | 14.768 | 0.271 | 0.9927 |

*Table S5b: Tukey’s post hoc test results of GLS regression for the rarefied diversity estimates (Hill’s numbers) corresponding to* ***q = 1 (Shannon index)****. For each pair-wise comparison (OG = old grassland; NG = new grassland; CN = cereal field near NG; CF = cereal field far from NG) the coefficient estimate, standard error, z-value and corresponding p-value is shown.*

| Comparison | Estimate | Std. Error | Z value | p-value |
| --- | --- | --- | --- | --- |
| CN-CF | 0.4320 | 0.2326 | 1.857 | 0.2281 |
| NG-CF | 0.7080 | 0.3081 | 2.298 | 0.0892 |
| OG-CF | 20.043 | 0.4778 | 4.195 | <0.001 |
| NG-CN | 0.2760 | 0.3641 | 0.758 | 0.8627 |
| OG-CN | 15.723 | 0.5157 | 3.049 | 0.0108 |
| OG-NG | 12.963 | 0.5539 | 2.341 | 0.0805 |

*Table S5c: Tukey’s post hoc test results of GLS regression for the rarefied diversity estimates (Hill’s numbers) corresponding to* ***q = 2 (Simpson index)****. For each pair-wise comparison (OG = old grassland; NG = new grassland; CN = cereal field near NG; CF = cereal field far from NG) the coefficient estimate, standard error, z-value and corresponding p-value is shown.*

| Comparison | Estimate | Std. Error | Z value | p-value |
| --- | --- | --- | --- | --- |
| CN-CF | 0.23300 | 0.15334 | 1.520 | 0.3974 |
| NG-CF | 0.30167 | 0.15056 | 2.004 | 0.1679 |
| OG-CF | 129.500 | 0.33260 | 3.894 | <0.001 |
| NG-CN | 0.06867 | 0.20658 | 0.332 | 0.9858 |
| OG-CN | 106.200 | 0.36143 | 2.938 | 0.0151 |
| OG-NG | 0.99333 | 0.36026 | 2.757 | 0.0256 |

**S6 – Ant community composition: PERMANOVA, homogeneity of dispersion**

*Table S6a. Results of PERMANOVA analysis of the effect of habitat type (OG = old grassland; NG = new grassland; CN = cereal field near NG; CF = cereal field far from NG) on ant community composition at transect level. Abbreviations: SS= sums of squares; MS= mean squares.*

| Source | df | SS | MS | pseudo-F | R² | p-value |
| --- | --- | --- | --- | --- | --- | --- |
| habitat type | 3 | 1.0799 | 0.35997 | 6.2473 | 0.48376 | 0.002 |
| Residuals | 20 | 1.1524 | 0.05762 |  | 0.51624 |  |
| Total | 23 | 2.2323 | 517.91 |  | 1.00000 |  |

*Table S6b. ANOVA results of homogeneity of dispersion tests of ant community composition among habitat types (OG = old grassland; NG = new grassland; CN = cereal field near NG; CF = cereal field far from NG). Abbreviations: SS= sums of squares; MS= mean squares.*

| Source | df | SS | MS | F-value | p-value |
| --- | --- | --- | --- | --- | --- |
| habitat type | 3 | 0.17621 | 0.058738 | 3.2524 | 0.043 |
| Residuals | 20 | 0.36120 | 0.018060 |  |  |

*Table S6c. Tukey’s post hoc results of homogeneity of dispersion tests of ant community composition among habitat types (OG = old grassland; NG = new grassland; CN = cereal field near NG; CF = cereal field far from NG).*

| Comparison | Estimate | Std. Error | Z value | p-value |
| --- | --- | --- | --- | --- |
| NG-OG | -0.12298141 | -0.3327826 | 0.086 | 0.379 |
| CN-OG | -0.15756021 | -0.3673614 | 0.052 | 0.186 |
| CF-OG | -0.21600996 | -0.4258111 | -0.006 | 0.042 |
| CN-NG | -0.03457879 | -0.2724710 | 0.203 | 0.976 |
| CF-NG | -0.09302855 | -0.3309207 | 0.144 | 0.696 |
| CF-CN | -0.05844976 | -0.2963419 | 0.179 | 0.900 |

**S7 – Functional trait space: PERMANOVA, homogeneity of dispersion**

*Table S7a. Results of PERMANOVA analysis of functional trait space of habitat types (OG = old grassland; NG = new grassland; CN = cereal field near NG; CF = cereal field far from NG). The analysis was based on an Euclidean distance matrix of the scaled and centered functional trait data (Table S1) of ant species present in the respective habitat types. Abbreviations: SS= sums of squares*

| Source | df | SS | R² | F | p-value |
| --- | --- | --- | --- | --- | --- |
| habitat type | 2 | 4.848 | 0.029 | 0.197 | 0.994 |
| Residuals | 13 | 160.152 | 0.971 |  |  |
| Total | 15 | 165.00 | 1 |  |  |

*Table S7b. ANOVA results of homogeneity of dispersion tests of ant functional trait space among habitat types (OG = old grassland; NG = new grassland; CN = cereal field near NG; CF = cereal field far from NG). The analysis was based on an Euclidean distance matrix of the scaled and centered functional trait data (Table S1) of ant species present in the respective habitat types. Abbreviations: SS= sums of squares; MS= mean squares.*

| Source | df | SS | MS | F-value | p-value |
| --- | --- | --- | --- | --- | --- |
| habitat type | 2 | 1.575 | 0.787 | 0.509 | 0.613 |
| Residuals | 13 | 20.113 | 1.547 |  |  |

**S8 – Biocontrol related functional traits: GLS results and Tukey’s post-hoc tests**

| *Table S8a: GLS regression results for prevalence of three functional traits in two habitats. Traits were chosen based on relevance for the provision of biocontrol services. Cereal field habitats (CF and CN) were excluded due to insufficient species richness. For the CWM values of each trait (CWM.Zoopha* *= assumed percentage of animal diet among total food intake, CWM.CS* *=colony size (ln transformed); CWM.FS* *= recruitment behaviour/foraging strategy) the coefficient estimate, standard error (in brackets) and t-value corresponding to each habitat type (OG = old grassland; NG = new grassland) is shown.* | | | |
| --- | --- | --- | --- |
|  | | | |
| *Predictor variable:* | *Response variable:* | | |
|  |  | | |
| Habitat type | CWM.Zoopha | CWM.FS | CWM.CS |
| NG | -0.040 | 0.034 | 0.015 |
|  | (0.196) | (0.056) | (0.123) |
|  | t = -0.206 | t = 0.606 | t = 0.120 |
| OG | -1.323^***^ | 0.477^***^ | 0.842^***^ |
|  | (0.150) | (0.055) | (0.110) |
|  | t = -8.835 | t = 8.673 | t = 7.684 |
|  | | | |
| Observations (df) | 14 | 14 | 14 |
| Log Likelihood | -5.141 | 11.669 | -2.009 |
| Akaike Inf. Crit. | 20.283 | -13.337 | 14.017 |
| Bayesian Inf. Crit. | 22.707 | -10.913 | 16.442 |
|  | | | |
| *Note:* | ^*^p^**^p^***^p<0.01 | | |

*Table S8b: Tukey’s post hoc test results of GLS regression for the prevalence of three functional traits in two habitats. For the CWM values of each trait (CWM.Zoopha* *= assumed percentage of animal diet among total food intake, CWM.CS* *=colony size (ln transformed); CWM.FS* *= recruitment behaviour/foraging strategy) the coefficient estimate, standard error and z- and p-value corresponding to each pairwise comparison among habitat types (OG = old grassland; NG = new grassland) is shown.*

| Comparison | Estimate | Std. Error | Z value | p-value |
| --- | --- | --- | --- | --- |
| CWM.Zoopha | -0.04039 | 0.19589 | -0.206 | 0.837 |
| CWM.FS | 0.00300 | 0.04772 | 0.063 | 0.95 |
| CWM.CS | 0.00739 | 0.12630 | 0.059 | 0.953 |

**S9 – Predation rates: GLS results**

| *Table S9: GLS regression results for the differences in predation rates corresponding to habitat type and mean vegetation cover. For each habitat type (OG = old grassland; NG = new grassland; CN = cereal field near NG; CF = cereal field far from NG) and respectively vegetation cover, the coefficient estimate, standard error, t-value and corresponding p-value is shown. Logit transformed predation rate on fruit flies served as response variable, habitat type and logit transformed mean vegetation cover of sampling plots served as predictor variables and study area (Elsbach, Ollern) served as random factor (via autocorrelation function).* | |
| --- | --- |
|  | |
| *Predictor variables:* | *Response variable:* |
|  |  |
| Habitat type * Vegetation cover | Predation rate |
|  | |
| CF | 1.848^***^ |
|  | (0.494) |
|  | t = 3.738 |
| CN | -1.648^**^ |
|  | (0.652) |
|  | t = -2.529 |
| NG | -2.517^**^ |
|  | (0.985) |
|  | t = -2.556 |
| OG | -2.694^*^ |
|  | (1.607) |
|  | t = -1.677 |
| Vegetation cover (CF) | -1.461^***^ |
|  | (0.307) |
|  | t = -4.759 |
| Vegetation cover (CN) | 0.995^**^ |
|  | (0.402) |
|  | t = 2.477 |
| Vegetation cover (NG) | 1.321^***^ |
|  | (0.422) |
|  | t = 3.130 |
| Vegetation cover (OG) | 1.607^***^ |
|  | (0.581) |
|  | t = 2.765 |
| Observations (df) | 90 |
| Log Likelihood | -110.547 |
| Akaike Inf. Crit. | 241.094 |
| Bayesian Inf. Crit. | 265.161 |
|  | |
| *Note:* | ^*^p^**^p^***^p<0.01 |

**S10– Predation rates: Tukey’s post-hoc tests**

*Table S10. Tukey’s post hoc test results of GLS regression for differences in predation rates between habitats. For each pair-wise comparison (OG = old grassland; NG = new grassland; CN = cereal field near NG; CF = cereal field far from NG) the coefficient estimate, standard error, t-value and corresponding p-value is shown. Logit transformed predation rate on fruit flies served as response variable, habitat type and logit transformed mean vegetation cover of sampling plots served as predictor variables and study area (Elsbach, Ollern) served as random factor (via autocorrelation function).*

| Comparison | Estimate | Std. Error | t value | p-value |
| --- | --- | --- | --- | --- |
| CN-CF | -0.472 | 0.391 | -1.206 | 0.6276 |
| NG-CF | -0.297 | 0.409 | -0.728 | 0.8854 |
| OG-CF | -0.730 | 0.580 | -1.258 | 0.5920 |
| NG-CN | 0.175 | 0.396 | 0.441 | 0.9708 |
| OG-CN | -0.258 | 0.577 | -0.446 | 0.9702 |
| OG-NG | -0.432 | 0.599 | -0.722 | 0.8882 |

**S11– Aboveground ant activity: GLS results and Tukey’s post-hoc tests**

| *Table S11a: GLS regression results for the differences in aboveground ant activity between habitats. For each habitat type (OG = old grassland; NG = new grassland; CN = cereal field near NG; CF = cereal field far from NG) the coefficient estimate, standard error (in brackets) and t-value is shown.* | |
| --- | --- |
| *Predictor variables:* | *Response variable:* |
|  |  |
| Habitat type | Ant activity |
|  | |
| CF | 2.686^***^ |
|  | (0.258) |
|  | t = 10.425 |
| CN | -0.140 |
|  | (0.366) |
|  | t = -0.382 |
| NG | 1.337^***^ |
|  | (0.357) |
|  | t = 3.745 |
| OG | 2.964^***^ |
|  | (0.321) |
|  | t = 9.226 |
|  | |
| Observations (df) | 84 |
| Log Likelihood | -114.646 |
| Akaike Inf. Crit. | 241.291 |
| Bayesian Inf. Crit. | 255.583 |
|  | |
| *Note:* | ^*^p^**^p^***^p<0.01 |

*Table S11b. Tukey’s post hoc test results of GLS regression for differences in aboveground ant activity between habitats. For each pair-wise comparison (OG = old grassland; NG = new grassland; CN = cereal field near NG; CF = cereal field far from NG) the coefficient estimate, standard error, t-value and corresponding p-value is shown. Tukey transformed number of observed workers served as response variable, habitat type served as predictor variables and study area (Elsbach, Ollern) served as random factor (via autocorrelation function).*

| Comparison | Estimate | Std. Error | t value | p-value |
| --- | --- | --- | --- | --- |
| CN-CF | 0.366 | 37.1 | 0.382 | 0.9808 |
| NG-CF | 0.357 | 34.7 | -3.745 | 0.0035 |
| OG-CF | 0.321 | 76.5 | -9.226 | <.0001 |
| NG-CN | 0.366 | 37.1 | -4.035 | 0.0014 |
| OG-CN | 0.324 | 79.0 | -9.574 | <.0001 |
| OG-NG | 0.321 | 76.5 | -5.066 | <.0001 |

**S12– Predatory groups observed during predation experiment**

*Table S12. Table showing the predator identity, the number of observations on cardboards during re-collection, the relative contribution of each predator to total number of observations (X out of 274 cardboards) and the contribution of each predator to total number of cardboards (X out 720 cardboards).*

| Predator identity | Observations (No. Of cardboards) | Contribution (relative; X out of 274) | Contribution (total, X out of 720) |
| --- | --- | --- | --- |
| Ants (Formicidae) | 138 | 50,36 % | 19,17 % |
| Carabid beetle (Carabidae) | 60 | 21,90 % | 8,33 % |
| Wasp (Hymenoptera) and/or Fly (Diptera) | 33 | 12,04 % | 4,58 % |
| Land slug (Gastropoda) | 23 | 8,39 % | 3,19 % |
| Spider (Araneae) | 18 | 6,57 % | 2,50 % |
| True bugs (Hemiptera) | 2 | 0,73 % | 0,28 % |
|  |  |  |  |
| Predator observed | 274 |  | 38,06 % |
| No predator observed | 446 |  | 61,94 % |
| Sum | 720 | 100,00 % | 100,00 % |
